# Denaturing gradient gel electrophoresis and multi-SIR profiles of soil microbial communities from a karst doline at Aggtelek National Park, Hungary

**DOI:** 10.1007/s12223-020-00828-y

**Published:** 2020-10-08

**Authors:** Márton Mucsi, Gergely Krett, Tibor Szili-Kovács, János Móga, Andrea K. Borsodi

**Affiliations:** 1 Institute for Soil Sciences and Agricultural Chemistry, Centre for Agricultural Research, Herman Ottó út 15, Budapest, H-1022 Hungary; 2 Department of Microbiology, ELTE Eötvös Loránd University, Pázmány Péter sétány 1/c, Budapest, H-1117 Hungary; 3 Department of Physical Geography, ELTE Eötvös Loránd University, Pázmány Péter sétány 1/c, Budapest, H-1117 Hungary; 4 Danube Research Institute, Centre for Ecological Research, Karolina út 29, Budapest, H-1113 Hungary

**Keywords:** Karst soil, Bacteria, DGGE, Multi-SIR, MicroResp

## Abstract

Soils play an important role in the ecosystem of karstic landscapes both as a buffer zone and as a source of acidity to belowground water. Although the microbiota of karstic soils is known to have a great effect on karstification processes, the activity and composition of these communities are largely unknown. This study gives a comparative analysis of soil microbial profiles from different parts of a doline located at Aggtelek, Hungary. The aim was to reveal the relationships between the vegetation type and genetic fingerprints and substrate utilisation (multi-SIR) profiles of the soil microbiota. Soil samples were collected in early and late springs along a transect in a doline covered with different types of vegetation. Genetic fingerprints of bacterial communities were examined by denaturing gradient gel electrophoresis (DGGE) based on the 16S rRNA gene, along with multi-SIR profiles of the microbial communities measured by the MicroResp method using 15 different carbon sources. Genetic fingerprinting indicated that vegetation cover had a strong effect on the composition of soil bacterial communities. Procrustean analysis showed only a weak connection between DGGE and multi-SIR profiles, probably due to the high functional redundancy of the communities. Seasonality had a significant effect on substrate usage, which can be an important factor to consider in future studies.

**Electronic supplementary material:**

The online version of this article (10.1007/s12223-020-00828-y) contains supplementary material, which is available to authorized users.

## Introduction

Karstic landscapes provide many ecosystem services, such as the production of drinking water for about 25% of the global population (Ford and Williams 2007). Also, because of their special microclimatic effects, karst dolines provide refugee to many vascular plants (Bátori et al. 2014). In the past few centuries, however, human activities had great impact on karstic ecosystems by influencing both karst forming processes and ecosystem services (Móga et al. 2013; Gutiérrez et al. 2014).

The microbial ecology of subsurface waters and caves, and the role of microbes in direct weathering of carbonate rocks and formation of minerals are intensively studied fields (Barton and Northup 2007; Lian et al. 2008; Baskar et al. 2016). Besides these, individual indicator organisms in karstic waters are also studied during the monitoring of human effects on karstic ecosystems (Mulec et al. 2012). The microbial ecology of epikarstic soils, however, is rarely included in the karst researches, despite the crucial role of soil microbiota playing in karstification processes.

The soil layer, when present, plays an important role as a buffer zone of water perturbations, as in most areas, the precipitation filtrates through the soil before reaching the subsurface aquifers (Williams 2008). Epikarstic soils are also hypothesised to have a huge impact on larger scale karst formation as the main source of acidity in subsurface waters (Williams 2008; Phillips 2016).

In Hungary, a few previous studies examined the relationship between karstic processes and soil microbiota. Bárány and Mezősi (1977) showed that in the case of dolines covered with grasslands, the number of culturable microbes in the upper 5 cm soil layer is mainly determined by the temperature, while in deeper layers, it is more related to soil moisture. That said, the number of microbes in different parts of a doline can be greatly influenced by the exposure, especially north vs south of the sampling site. It was also shown that microbial strains from terra rossa type soil types can have higher limestone corrosion abilities, than microbes from rendzinas (Darabos 1999).

Soil pH was recently shown to be the main factor in shaping the composition of belowground bacterial communities of karstic areas (Yun et al. 2016). Different vegetation types, however, are also known to correspond with belowground microbial populations (Hooper et al. 2000), mainly through the composition of plant litter and root exudates (Berg and Smalla 2009). Besides this, Kevei and Zámbó (1985) showed that the density of aerobic culturable bacteria is higher in dolines covered with forests, possibly because of more stable microclimatic – soil moisture and temperature – conditions.

In another study, soil microbial communities from two different Hungarian karst areas were compared by substrate induced respiration (SIR) and genetic fingerprinting by denaturing gradient gel electrophoresis (DGGE) (Knáb et al. 2012). The results showed that the structure of soil bacterial communities were clearly distinct in different sampling sites; however, microbial respiration rates only slightly differed in the top soil but more influenced by the soil depth.

Beside the widespread study of soil microbial community composition, in the last decade, the research of catabolic processes using fingerprinting methods also became a widely used in the assessment of soil functioning and in soil monitoring (Wakelin et al. 2013; Nazaries et al. 2015; Creamer et al. 2016). However, these methods have been rarely applied to epikarstic soils.

Therefore, this study aimed to reveal differences in the DGGE and multi-SIR profiles of soil microbial communities from a typical karst doline in the Aggtelek National Park, Hungary. The relationship between soil properties, vegetation cover and microbiota was investigated, as well as possible correlations between genetic and catabolic fingerprints of the communities. Seasonal changes of the community fingerprints were also assessed.

## Materials and methods

### Study site and sampling

**Fig. 1:**
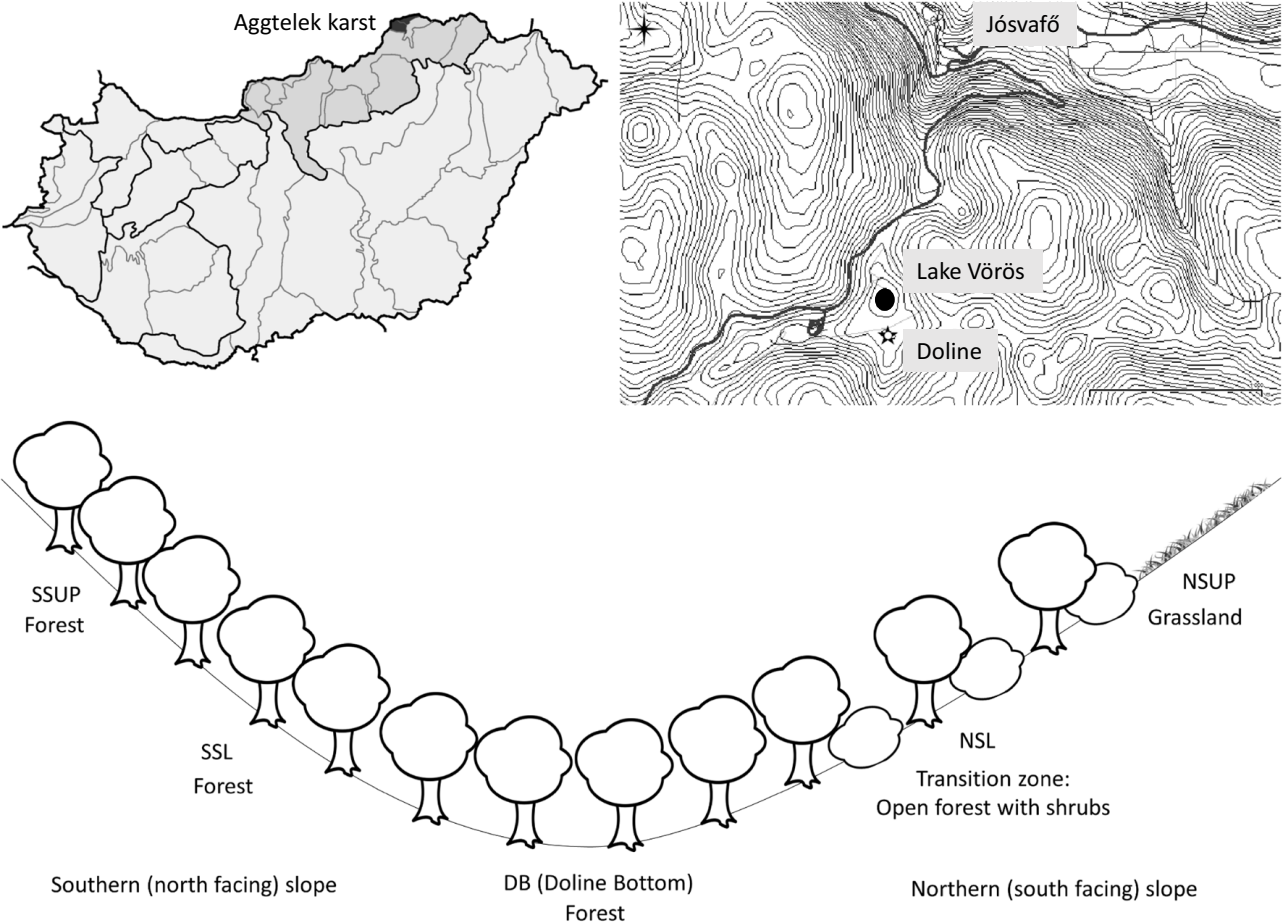
Location of the sampling area and vegetation cover in the Lake Vörös doline (Aggtelek National Park, Hungary)

The doline is located in the karst of Aggtelek National Park, near Lake Vörös (48.4715° N, 20.5426° E, 326 m above sea level, Fig. 1). As a result of reforestation, the southern part and the bottom of the doline is now covered by forest dominated by *Acer campestre* and *Carpinus betulus* without significant understory. On the northern slope, the forest is gradually transitioned into diverse grassland which is maintained by mowing and chopping of shrubs, resulting in a mosaic forest-steppe vegetation (see Online Resource 1–5 for photos of the vegetation at the sampling sites). The soils in the doline were identified according to the Hungarian Soil Classification System by Kiss (2012) as brown forest soil with clay illuviation at southern slope, red clay rendzina at northern slope and slope sediment soil at the bottom of the doline, all three developed on red clay. The former two can be classified in the WRB system as leptic Luvisols (clayic, chromic) and the soils from the doline bottom as Regosols.

The samplings were performed at the early (April) and late (June) spring period. Five sampling sites were set in north-south direction, with two sampling sites on the southern slope, one at the bottom of the doline and further two on the northern slope (Fig. 1). Triplicate soil samples from each site were collected by spade from the 0–10 cm layer of 1 m × 1 m quadrates (15 samples altogether). Samples were stored in PE zip-lock bags at 4 °C until the physical-chemical and catabolic experiments. Approximately 10 g soil of each sample was immediately put in sterile tubes and was stored at − 20 °C for molecular analysis.

### Soil physical and chemical properties

Soil water content was determined by drying 15 g of each sample at 105 °C. pH-KCl and pH-H_2_O were determined from 1:2.5 suspensions of soil:KCl and soil:H_2_O, respectively. Total organic carbon (C_org_) content, total salt content (%) from electrical conductivity, and soil texture were also determined. The above measurements were done by the Hungarian standard methodology (Buzás 1988, 1993).

### DNA extraction and PCR-DGGE

Total soil community DNA was extracted from approximately 0.25 g soil of each sample using PowerSoil DNA Isolation Kit (MO BIO Laboratories Inc., Carlsbad, CA, USA). The V1 region of the bacterial 16S rRNA gene was amplified by two consecutive PCR (Polymerase Chain Reaction), using first the primers 27F (Lane 1991) and 1401R (Nübel et al. 1996) and a GC-clamp 27F-GC and 519R (Turner et al. 1999) primers thereafter. The PCR mixtures contained 2 μL of purified genomic DNA, 0.2 mM of each deoxynucleotide, 2 mM MgCl_2_, 1 U LC *Taq* DNA Polymerase (Fermentas, Vilnius, Lithuania), 1× PCR Buffer (Fermentas, Vilnius, Lithuania) and 0.325 μM of the primers in a final volume of 50 μL. Temperature profile of both PCRs included an initial denaturation at 95 °C for 5 min, followed by 32 cycles (denaturation at 94 °C for 30 s, annealing at 52 °C for 30 s, extension at 72 °C for 1 min) and a final extension at 72 °C for 10 min. The DNA content of the samples was checked by electrophoresis in 1% agarose gel after each step.

DGGE was carried out using an INGENY phorU gel electrophoresis apparatus (Ingeny International BV, Goes, Netherlands), at 60 °C and a charge of 120 V for 14 h in 7% polyacrylamide gel containing 40 to 60% gradient of denaturants (100% was defined as 40% formamide and 7 M urea). The gel was stained by ethidium bromide for 20 min, then the patterns were visualized by an UV transilluminator. The patterns were digitalised for later analysis by taking a photograph.

### Soil multi-SIR profiles

Measurements were done with the MicroResp system (Campbell et al. 2003), using the protocol provided by the manufacturer (MSC Ltd., Aberdeen, UK). Soil samples were filled into deep-well microplates (one sample/plate) and covered by Parafilm. They were then put into a desiccator together with a wet towel and sodalime for a 5-day preincubation at room temperature. For the measurements, 15 carbon sources (D-glucose, trehalose, D-galactose, L-arabinose, D-fructose, citric acid, malic acid, Na-succinate, L-arginine, L-alanine, L-leucine, L-lysine, L-glutamine, L-glutamic acid and 3,4-dihydroxybenzoic acid) were used in 6 repetitions on each plate, with distilled water as control. After substrate addition, a 5 h incubation period at 25.2 °C was applied; then, substrate utilization patterns were measured by a microplate reader (Anthos 2010, Biochrom, Cambridge, UK) as absorbance changes of the gel in the detector plates. Absorbance values measured at 570 nm were converted into %CO_2_ values using the equation given by the manufacturer (%CO_2_ = A + B / (1 + D × Ai), where A, B and D are constants and Ai are the individual absorbance values after incubation). Detector plates were calibrated on different CO_2_ concentrations using a gas chromatograph (Fisons GC 8000) prior to the experiment to determine the values of the A, B and D constants. %CO_2_ values were then converted into CO_2_ production rates (μgCO_2−_C × g soil^−1^ × h^−1^) using the calculation given by the manufacturer.

### Statistical analysis

DGGE patterns were analysed using the TotalLab 120 software (TotalLab LTD., UK). Bands were detected automatically after subtracting the background level with the rolling ball method. Bands were grouped automatically based on their position in the lanes. Automatic detection and grouping were then manually checked for errors, e.g. impurities in the gel detected as bands. Similarity matrices were created on the basis of presence or absence of bands, then dendrograms were generated using the UPGMA method. Raw presence-absence data was then exported for further analysis with the R 3.5.0 (R Core Team 2018). Bands that had zero variance along the samples were removed from the data sets, then principal components analysis (PCA) was applied on the centred data matrix, using the prcomp function of the vegan package (Oksanen et al. 2018). Visualization of the ordinations was done using the ggbiplot package (Vu 2011) with custom modifications for visuals. The ordinations from the two seasons were compared based on the first two principal components, determined by the broken stick method. For the comparison of the two season, symmetric Procrustes test (Peres-Neto and Jackson 2001) was applied using the protest function, with 999 permutations to test the significance of the statistic.

Data from MicroResp were analysed using MS Excel and R. Raw data were processed as described in the MicroResp protocol to determine CO_2_ production rates (μg CO_2_-C × g soil^−1^ × h^−1^) for each well. Respiration data were standardized by the average respiration rate for each plate. PCA was carried out on the data from the two seasons separately; then, Procrustes test was applied similarly as before, to detect significant differences between the two sampling times. For testing the differences between locations, PERMANOVA was applied with different a priori sample groups, using Bray-Curtis index and 9999 permutations (Anderson 2001).

To determine how well the genetic and catabolic fingerprints correlate, the ordinations from DGGE and MicroResp data were also compared by Procrustes tests.

## Results

### Soil physical and chemical properties

Soils were slightly acidic, with high organic C content (Table 1.). Soil textures varied between silty loam, silty clay loam and silty clay on different parts of the doline. While the samples from the two slopes had similar texture, the doline bottom had much higher proportion (over 71%) of silt and more variability in the pH. It can be because soils from the slopes were redepositioned by rainfall erosion, resulting in a mixed sediment at the doline bottom which is a common phenomenon (Kiss 2012).

**Table 1:** Physical and chemical properties of the soil samples from the Lake Vörös doline (Aggtelek National Park, Hungary). *SS*, southern slope; *NS*, northern slope, W% is the gravimetric water content in percentage of total soil weight

Sample	Location	Vegetation	pH_H2O_	pH_KCl_	Organic C content (m/m%)	total salt content (m/m%)	W% (late spring)	W% (early spring)	Texture
SSUP1	SS, upper part	Closed canopy forest	6.33	5.53	3.61	0.03	30.30	35.69	Silty clay loam
SSUP2	SS, upper part	Closed canopy forest	6.46	5.90	5.39	0.04	33.62	34.35	Silty clay loam
SSUP3	SS, upper part	Closed canopy forest	5.80	4.98	3.85	< 0.02	30.16	34.16	Silty clay loam
SSL1	SS, lower part	Closed canopy forest	5.53	4.46	3.66	0.04	28.36	34.88	Silty clay loam
SSL2	SS, lower part	Closed canopy forest	5.27	4.20	2.89	< 0.02	25.16	35.31	Silty clay loam
SSL3	SS, lower part	Closed canopy forest	5.59	4.65	3.44	0.04	29.90	33.37	Silty clay loam
DB1	Doline bottom	Closed canopy forest	4.31	3.38	3.55	< 0.02	28.01	29.83	Silt loam
DB2	Doline bottom	Closed canopy forest	5.79	4.99	2.76	< 0.02	27.36	31.84	Silt loam
DB3	Doline bottom	Closed canopy forest	6.08	5.20	3.21	0.04	28.13	31.65	Silt loam
NSL1	NS, lower part	Open canopy forest	6.61	5.79	3.75	0.06	28.83	25.56	Silty clay loam
NSL2	NS, lower part	Open canopy forest	6.00	5.04	3.06	< 0.02	28.48	28.13	Silty clay loam
NSL3	NS, lower part	Open canopy forest	6.12	5.18	3.11	0.04	28.14	29.79	Silty clay loam
NSUP1	NS, upper part	Grassland	5.80	4.90	3.31	< 0.02	27.64	25.72	Silty clay
NSUP2	NS, upper part	Grassland	5.85	4.94	2.91	< 0.02	27.02	19.6	Silty clay
NSUP3	NS, upper part	Grassland	6.01	4.97	2.69	< 0.02	25.60	24.88	Silty clay

### DGGE profiles of bacterial communities

**Fig. 2:**
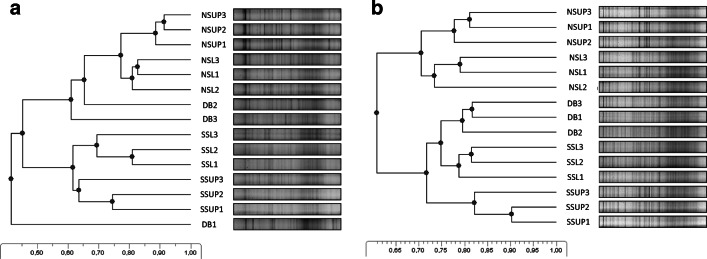
UPGMA dendrograms created from the DGGE patterns of soil bacterial communities in the late spring (**a**) and early spring (**b**) samples. Scales show the similarity of the samples between a range of 0–1

Dendrograms based on the DGGE profiles were similar in the two sampling times but some differences occurred (Fig. 2a and b). From the DB1 sample in late spring, only low amounts of DNA could be extracted thus it was placed in outsider position on the dendrogram. The DB samples clustered with the northern slope of the doline (NSUP and NSL) in the early spring, and with the southern slope (SSUP and SSL) in the late spring. Sites with different vegetation cover—forest, open forest, and grassland—had clearly distinct bacterial communities in both samplings.

**Fig. 3:**
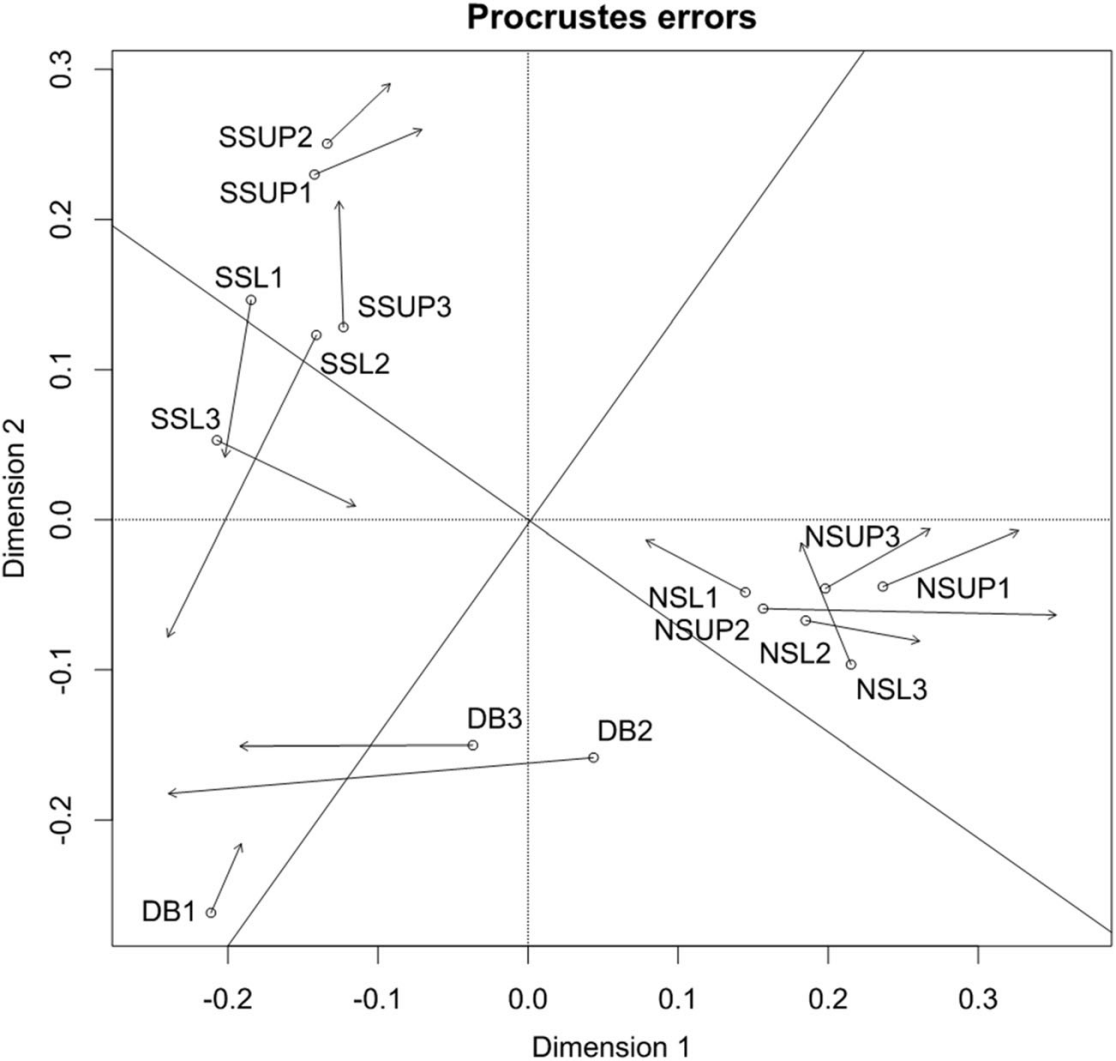
Procrustes analysis of the ordinations of DGGE data from the two sampling times. *r* = 0.855, *m*^2^ = 0.269, *p* = 0.001. Arrows are directed from the early to the late spring positions

The PCA of the data confirmed that the microbial community profiles were different in the five parts of doline, and showed that doline bottom has higher variance, probably because of the slope sediment nature of this area. Temporal differences can also be observed in the relative position of the samples (Online Resource 6 a and b). However, Procrustes test (Fig. 3) indicated that despite the different clustering of the doline bottom samples, the DGGE profiles of the microbial communities was quite similar in the two sampling times (*r* = 0.855, *p* = 0.001).

### Multi-SIR profiles

**Fig. 4:**
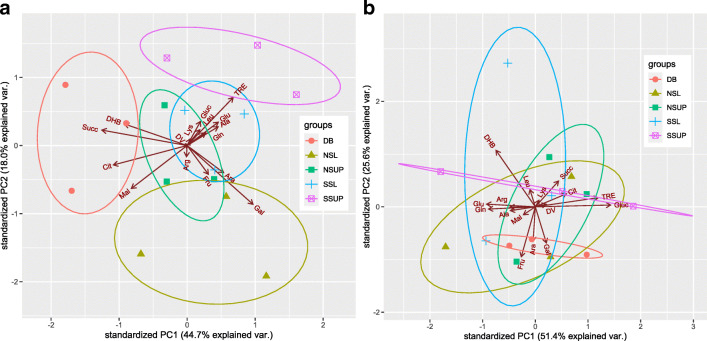
Results of principal components analysis of MicroResp fingerprints from early (**a**) and late (**b**) spring period. Ellipses represent the 95% confidence intervals of the groups. Arrows of the biplot correspond to the contribution of the original substrates to the variance represented by the principal components

The average respiration rates of the samples were similar in the two sampling times but showed a high variability between parallel samples. The PCA could not reveal differences among the substrate usage patterns of the microbial communities in the late spring but it could separate some of the sample groups in the early spring (Fig. 4a and b). Procrustes test showed that the catabolic fingerprints were very different in the two sampling times (*r*^2^ = 0.272, *p* = 0.581). The PCA of the combined dataset from the two sampling times (Online Resource 4) showed that the main difference was along PC 1, which had strong correlation (|*r*| > 0.6) with three of the organic substrates (malic acid, lysine and leucine), among which malic acid had the highest loading value (0.94). Early spring samples had higher respiration rates for malic acid (1.32 ± 0.52 μg CO_2_-C × g soil^−1^ × h^−1^ for late spring, vs. 5.5 ± 1.33 μg CO_2_-C × g soil^−1^ × h^−1^ for early spring).

Although not clearly separated by PCA, further analysis of the samples by PERMANOVA revealed that in the early spring, samples from different parts of the doline had distinct multi-SIR profiles. This difference was the most pronounced (*p* = 0.0004, pseudo-*F* = 3.96, all pairwise *p*-values below 0.05) when the samples were classified into three groups by their geographical location as northern slope, doline bottom, and southern slope. In the late spring, however, no statistically significant difference was found between samples.

Comparisons of MicroResp and DGGE fingerprints differed in the two sampling times. While in the early spring period there was a moderate Procrustean correlation between the two fingerprints (*r*^2^ = 0.605, *p* = 0.004), there was no significant correlation (*r*^2^ = 0.187, *p* = 0.848) between the two fingerprints in the late spring period.

## Discussion

Soil microbial density and diversity are known to be influenced by many factors. It was shown recently that in a karstic ecosystem, soil pH is the main parameter influencing the microbial communities (Yun et al. 2016). Although the method used in the present study is not suitable for detailed and quantitative analysis of community structure, and hence a direct comparison with the results of Yun et al. (2016) is not possible, DGGE is an excellent tool for comparative analysis of DGGE profiles (Nielsen et al. 2013), and is often used to indicate changes in the bacterial community structure after soil treatment or different land uses (e.g. Stagniari et al. 2014; Orlewska et al. 2018a, b). In our study, pH was not different significantly in different parts of doline—slightly acidic through the whole transect—still, there were significant differences in the genetic fingerprints of bacterial communities.

It is also known that vegetation can have a strong influence on soil microbial communities (Hooper et al. 2000; Haichar et al. 2014; Khlifa et al. 2017). Our results are similar to those of Li et al. (2014), who found in that different soil microbial communities developed at different successional stages of a karstic. In our study, areas with different vegetation type had clearly different bacterial communities. Plant communities can also affect soil microbes through changes in microclimatic relations. It was previously that areas covered by forests had much higher bacterial counts than those covered by grassland in karst dolines, because of more stable water conditions (Kevei and Zámbó 1985). Zhang et al. (2006) also found that in karstic areas, DGGE fingerprints, basal respiration and SIR rates better reflected the changes in plant coverage and vegetation type than the soil biochemical properties which is in good agreement with our results.

In the case of catabolic activities, however, we found that the relationship between vegetation and multi-SIR profiles was much weaker. In the late spring period, we could not indicate any differences in the substrate usage of microbial communities. In the early spring period, catabolic fingerprints were different on the two slopes and the bottom of the doline, but unlike in the case of DGGE profiles, no further distinction were possible e.g. between shrubs and grassland vegetation. The northern slope was slightly drier in this season; therefore, it is possible that the slightly different soil moisture played a role in the better separation of the samples, because it can have an effect on microbial communities (Kevei and Zámbó 1985).

There were, however, significant differences between the two sampling times, especially in the ability of microbial communities to utilise malic acid. It is already known that microbial communities are affected by root exudates (Haichar et al. 2014), which can possibly result in changes in catabolic fingerprints. Also, plant roots are known to actively exudate malate—along with citrate—to increase phosphate availability in the rhizosphere (Jones 1998), and also to recruit beneficial bacteria from the soil by malic acid exudation (Berendsen et al. 2012). Although drawing conclusions regarding root exudates is beyond the scope of this study, it is possible that the observed differences of catabolic fingerprints are a result of vegetation changes during the seasons. Nevertheless, seasonality clearly has a great effect on soil multi-SIR profiles. This effect is surprisingly rarely addressed in the literature, even in areas other than karst research, but it should definitely be taken into consideration in future studies.

We found that the correlation between genetic and catabolic fingerprints was weak in the early spring, and not significant in the late spring. This, together with the high number of bands in DGGE fingerprints, suggests that karstic soils can have very high microbial diversity and that it can result in significant redundancy of soil functional—in this case, catabolic—diversity (Wertz et al. 2006; Nielsen et al. 2011). Because of this, changes in bacterial community structure might be masked by functional redundancy which might limit the use of MicroResp method in itself for soil monitoring. Our results are in accordance with the study of Zhu et al. (2012), who examined a karstic area with five different vegetation succession stages, and found that DGGE was more sensitive than catabolic fingerprinting.

## Conclusions

Our study showed that vegetation differences can have a significant effect on the genetic fingerprint of soil microbial communities in a karst doline. However, these differences are not necessarily reflected in the catabolic activity and diversity because of the high functional redundancy of these soils. Our results also confirmed that seasonality can have a significant effect on the multi-SIR profiles of microbial communities. These effects can have a great influence on the results of soil monitoring and assessment of soil functions and should be taken into consideration in further studies.

## Electronic supplementary material

ESM 1 Vegetation of the SSUP sampling sites on the southern slope (early spring). (JPG 3717 kb)

ESM 2 Vegetation of the SSL sampling sites on the southern slope (early spring). (JPG 3855 kb)

ESM 3 Vegetation of the DB sampling sites on the doline bottom (early spring). (JPG 3734 kb)

ESM 4 Vegetation of the NSL sampling sites (shrubs) on the northern slope from above (late spring). Due to dense vegetation we could not take informative photos on the site itself. (JPG 4577 kb)

ESM 5 Vegetation of the NSUP (grassland) sampling sites on the northern slope (late spring). (JPG 4684 kb)

ESM 6 Results of Principal Components Analysis of DGGE fingerprints from early (a) and late (b) spring period. Ellipses represent the 95% confidence intervals of the groups. (PPTX 67 kb)

ESM 7 Principal components analysis of the MicroResp data for the early spring (group of point on the right), and late spring (group pf points on the left) samples. Contribution of the original substrates to the variance represented by the principal components are shown as arrows of the biplot. (PPTX 56 kb)

## Data Availability

Not applicable.
