# Supplementary figures and images for: Denaturing gradient gel electrophoresis and multi-SIR profiles of soil microbial communities from a karst doline at Aggtelek National Park, Hungary
Source: Folia Microbiol (Praha). 2020 Oct 8;66(1):107–14. doi: 10.1007/s12223-020-00828-y (PMC7854432; doi:10.1007/s12223-020-00828-y)

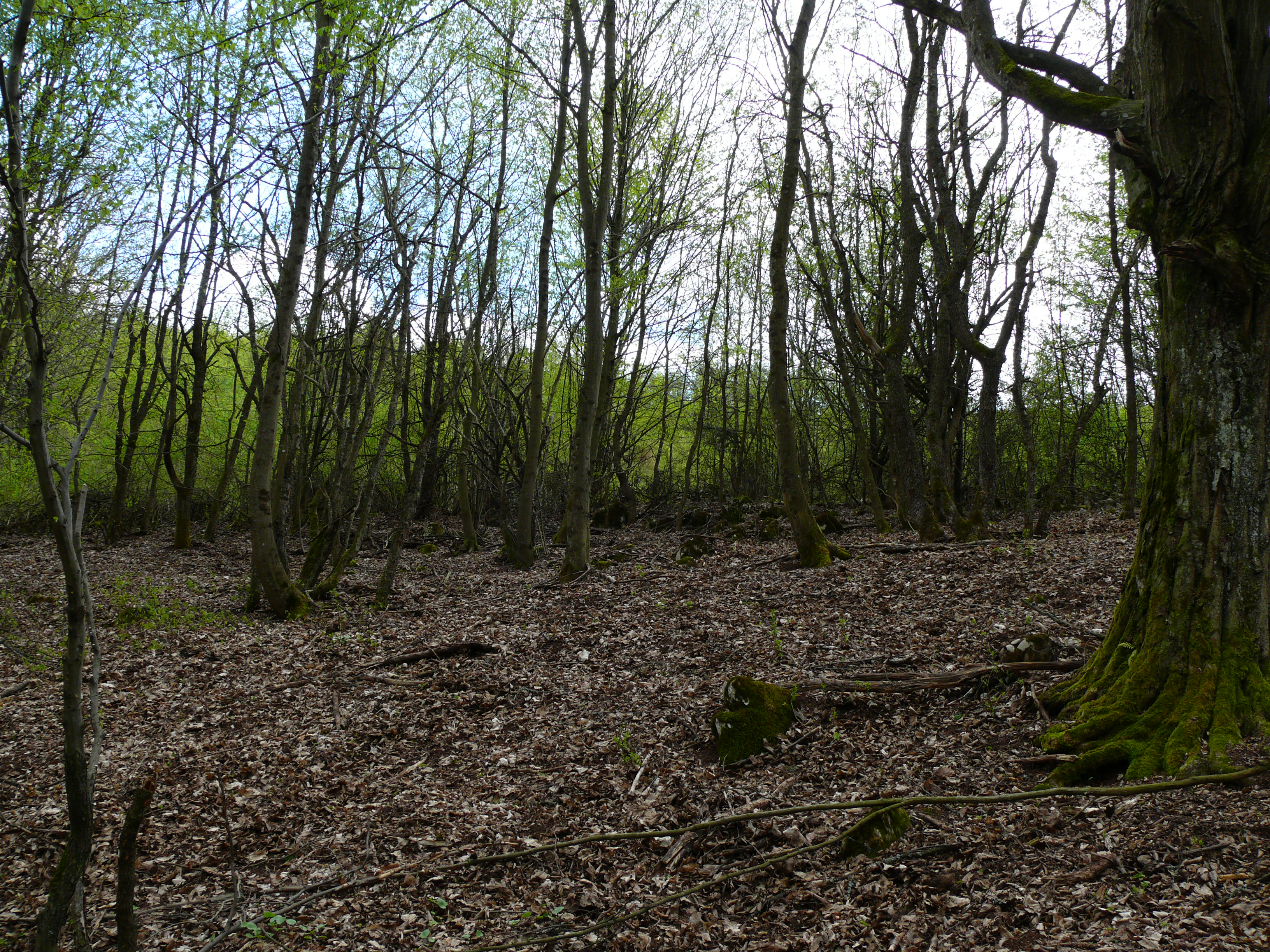

Supplement: Supplementary file 1 — Vegetation of the SSUP sampling sites on the southern slope (early spring). (JPG 3717 kb) [file 12223_2020_828_MOESM1_ESM.jpg]

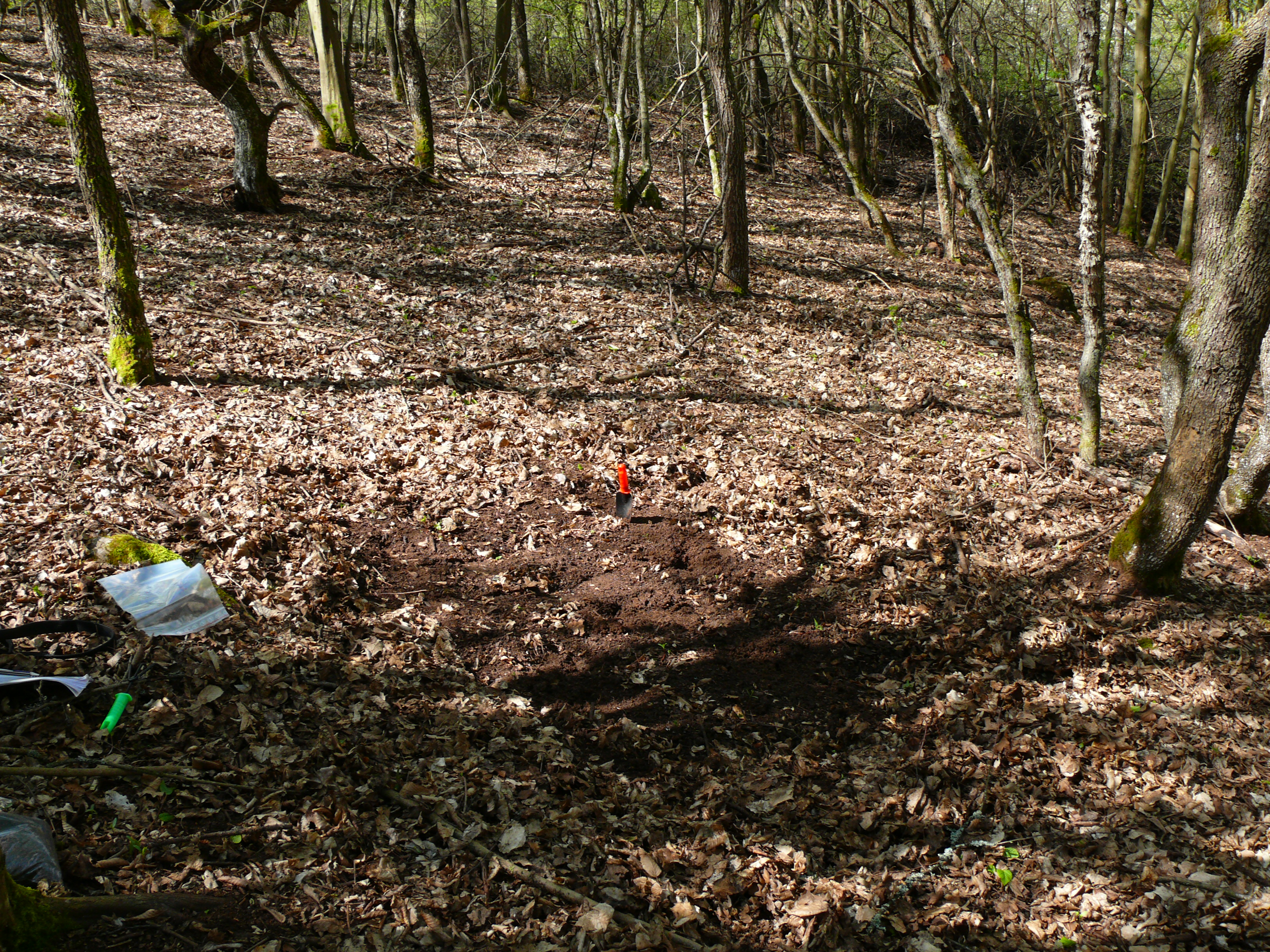

Supplement: Supplementary file 2 — Vegetation of the SSL sampling sites on the southern slope (early spring). (JPG 3855 kb) [file 12223_2020_828_MOESM2_ESM.jpg]

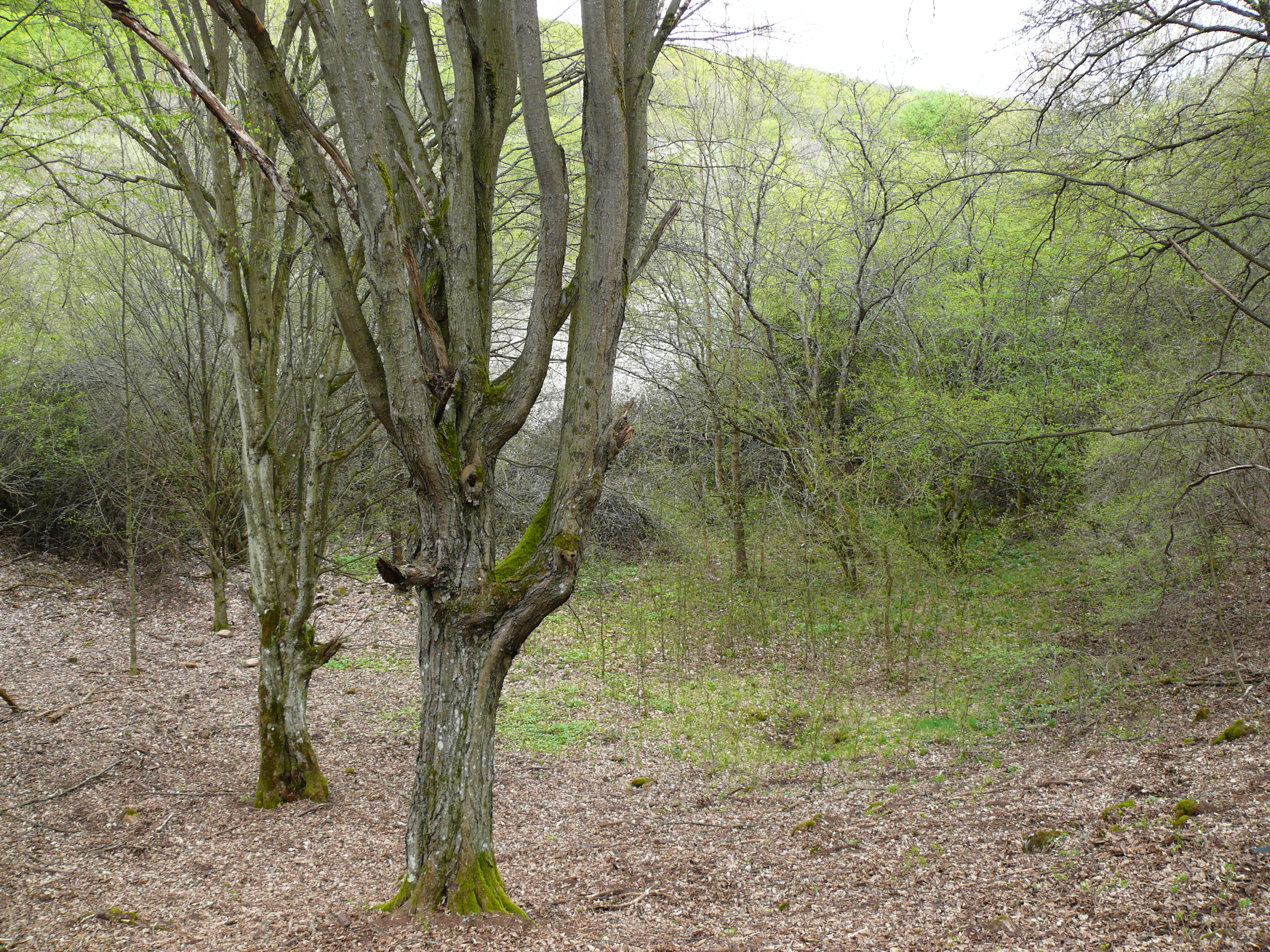

Supplement: Supplementary file 3 — Vegetation of the DB sampling sites on the doline bottom (early spring). (JPG 3734 kb) [file 12223_2020_828_MOESM3_ESM.jpg]

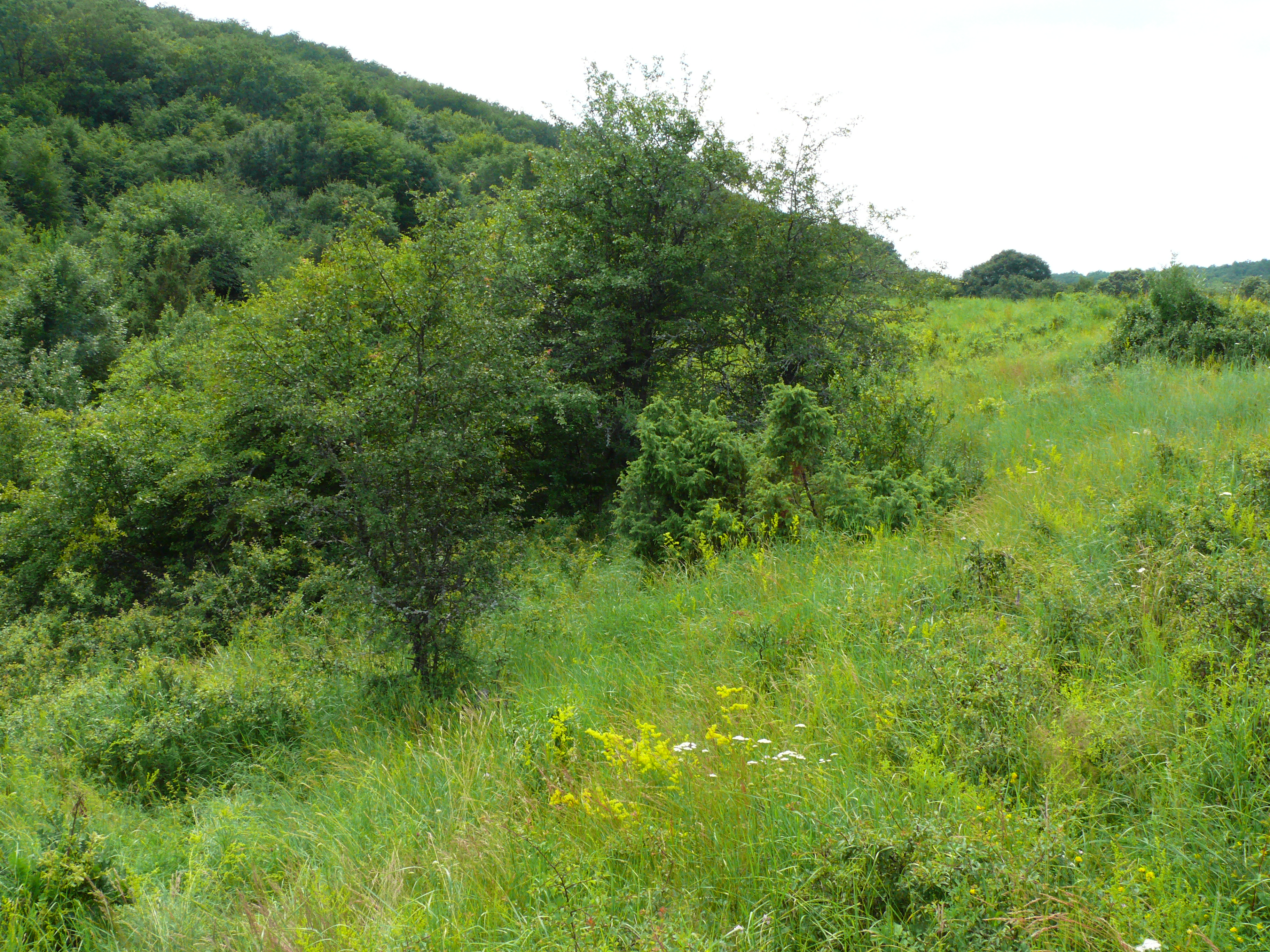

Supplement: Supplementary file 4 — Vegetation of the NSL sampling sites (shrubs) on the northern slope from above (late spring). Due to dense vegetation we could not take informative photos on the site itself. (JPG 4577 kb) [file 12223_2020_828_MOESM4_ESM.jpg]

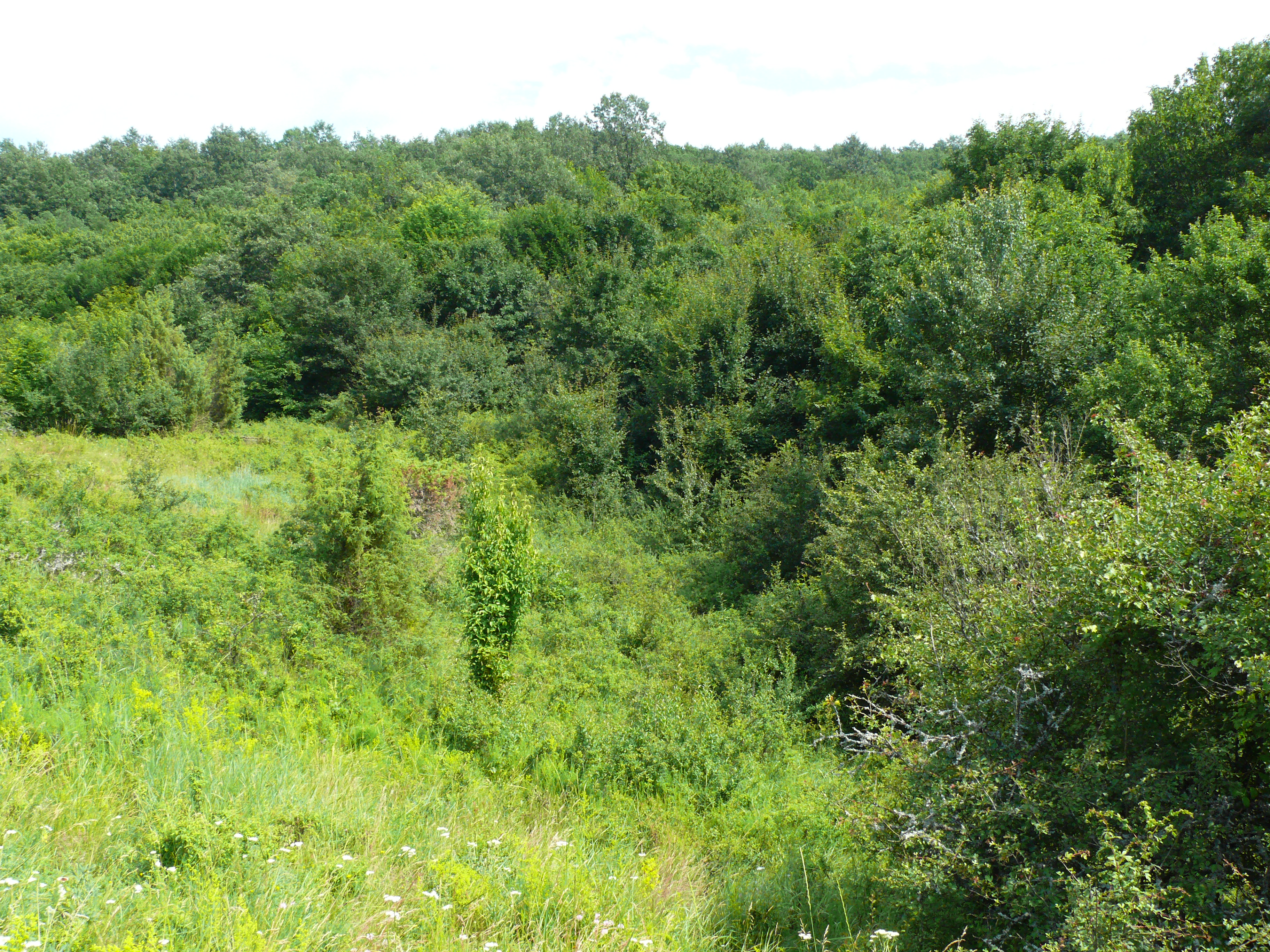

Supplement: Supplementary file 5 — Vegetation of the NSUP (grassland) sampling sites on the northern slope (late spring). (JPG 4684 kb) [file 12223_2020_828_MOESM5_ESM.jpg]

## Slide 1
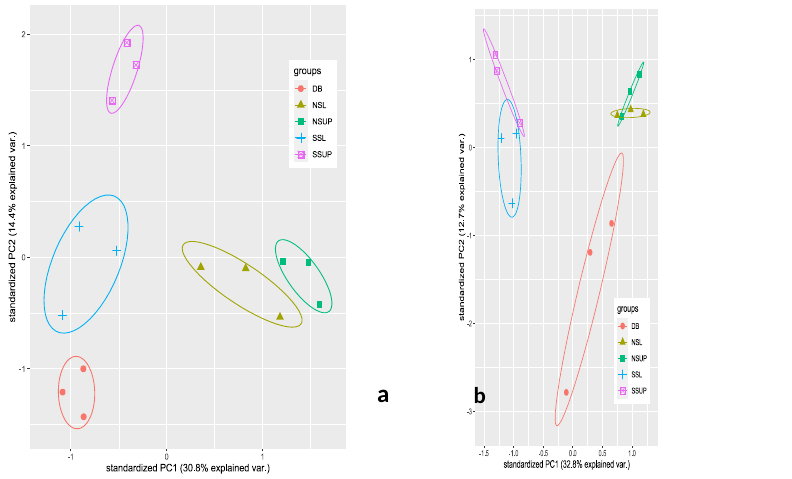

a
b

Supplement: Supplementary file 6 — Results of Principal Components Analysis of DGGE fingerprints from early (a) and late (b) spring period. Ellipses represent the 95% confidence intervals of the groups. (PPTX 67 kb) [file 12223_2020_828_MOESM6_ESM.pptx]

## Slide 1
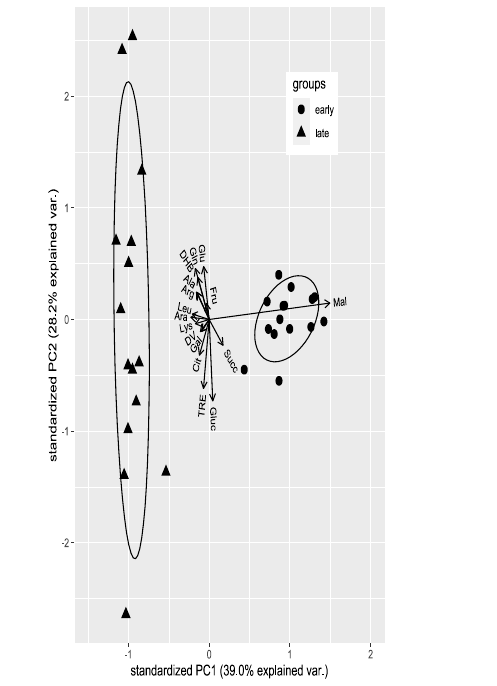

Supplement: Supplementary file 7 — Principal components analysis of the MicroResp data for the early spring (group of point on the right), and late spring (group pf points on the left) samples. Contribution of the original substrates to the variance represented by the principal components are shown as arrows of the biplot. (PPTX 56 kb) [file 12223_2020_828_MOESM7_ESM.pptx]
